# Concurrent Learning Approach for Estimation of Pelvic Tilt from Anterior–Posterior Radiograph

**DOI:** 10.3390/bioengineering11020194

**Published:** 2024-02-17

**Authors:** Ata Jodeiri, Hadi Seyedarabi, Sebelan Danishvar, Seyyed Hossein Shafiei, Jafar Ganjpour Sales, Moein Khoori, Shakiba Rahimi, Seyed Mohammad Javad Mortazavi

**Affiliations:** 1 Faculty of Electrical and Computer Engineering, University of Tabriz, Tabriz 51666, Iran; a.jodeiri@tbzmed.ac.ir; 2 Faculty of Advanced Medical Sciences, Tabriz University of Medical Sciences, Tabriz 51656, Iran; 3 College of Engineering, Design and Physical Sciences, Brunel University London, Uxbridge UB8 3PH, UK; 4 Orthopedic Surgery Research Centre, Sina University Hospital, School of Medicine, Tehran University of Medical Sciences, Tehran 51656, Iran; 5 Department of Orthopedic Surgery, Shohada Hospital, Tabriz University of Medical Sciences, Tabriz 51656, Iran; 6 Joint Reconstruction Research Center (JRRC), Tehran University of Medical Sciences, Tehran 51656, Iran; moein.khoori@gmail.com (M.K.); smjmort@yahoo.com (S.M.J.M.)

**Keywords:** total hip arthroplasty, pelvic tilt, multi-task learning, convolutional neural network, segmentation, VGG, U-NET

## Abstract

Accurate and reliable estimation of the pelvic tilt is one of the essential pre-planning factors for total hip arthroplasty to prevent common post-operative complications such as implant impingement and dislocation. Inspired by the latest advances in deep learning-based systems, our focus in this paper has been to present an innovative and accurate method for estimating the functional pelvic tilt (PT) from a standing anterior–posterior (AP) radiography image. We introduce an encoder–decoder-style network based on a concurrent learning approach called VGG-UNET (VGG embedded in U-NET), where a deep fully convolutional network known as VGG is embedded at the encoder part of an image segmentation network, i.e., U-NET. In the bottleneck of the VGG-UNET, in addition to the decoder path, we use another path utilizing light-weight convolutional and fully connected layers to combine all extracted feature maps from the final convolution layer of VGG and thus regress PT. In the test phase, we exclude the decoder path and consider only a single target task i.e., PT estimation. The absolute errors obtained using VGG-UNET, VGG, and Mask R-CNN are 3.04 ± 2.49, 3.92 ± 2.92, and 4.97 ± 3.87, respectively. It is observed that the VGG-UNET leads to a more accurate prediction with a lower standard deviation (STD). Our experimental results demonstrate that the proposed multi-task network leads to a significantly improved performance compared to the best-reported results based on cascaded networks.

## 1. Introduction

The pelvic bone, as a connection between the axial skeleton and lower limbs, plays a significant role in standing balance [1]. Nonetheless, the acetabular component position in total hip arthroplasty is affected by changes in the pelvic orientation during daily activities. The pelvic tilt is the primary representative of pelvic orientation [2]. To accomplish satisfactory outcomes and prevent postoperative complications after total hip replacement, including dislocation, impingement, and abnormal wear, it is crucial to accurately measure pelvic tilt angles [3,4,5]. The appropriate pelvic tilt is less than 20 degrees [6]. An increase in this parameter is considered abnormal pelvic and spinal sagittal balance. Based on the mentioned statement, it is essential to evaluate the pelvic tilt for better diagnosis and surgical planning in spine and hip surgery. The current method is an operator-dependent measurement on a lateral spinopelvic X-ray, which has at least two drawbacks: first, performing a lateral spinopelvic radiograph in addition to an anteroposterior pelvic X-ray that is routinely performed for these patients, and next, its operator-dependent nature [7].

The modern definition of pelvic tilt contains the anterior pelvis plane (APP), which is the line between two anterior superior iliac spines (ASIS) and the pubic tubercle. The pelvic tilt is then defined as the angle between the APP and the coronal plane, which is any vertical plane that divides the body into ventral and dorsal sections [8,9]. The other definition that is widely used is an angle between two lines, first a line that connects the femoral head to the center of the first sacral vertebra (S1) superior endplate, and the second line is a line perpendicular to the femoral head [10]. The latter method is more practical than the former but pointing to the center of the femoral head is not easy, as two femoral heads will not exactly superimpose to each other on a lateral spinopelvic X-ray; in such a situation, the surgeons connect two femoral heads and assume the center of this line to be the center of the femoral head. Currently, preoperative standing lateral radiographs are routinely conducted to calculate pelvic tilt. Although their accuracy in comparison to three-dimensional techniques is less, they are widely used due to their availability and low expenses. However, accurate interpretation of the X-rays is not only complicated but also operator-dependent [7].

Currently, the technique used for measuring pelvic tilt is based on obtaining a lateral lumbopelvic radiograph in a standing position and occasionally a sitting position to consider the differences that might happen, such as increasing anteversion in the sitting position [11]. In the conventional method for pelvic tilt measurement from a lateral X-ray, we estimate the pelvic tilt from the angle created between the line connecting the center of the femoral head to the midpoint of the superior endplate of the S1 and the vertical reference line [12]. These lateral lumbopelvic radiographs, however, focus mainly on the pelvis and do not demonstrate the entire lumbar vertebra, so they do not have a considerable clinical application except for the estimation of the pelvic tilt.

Therefore, if we can measure the pelvic tilt based on our proposed method from a standing AP X-ray with acceptable/reasonable accuracy, we will be able to estimate the pelvic tilt with an image that presently is not applied to measure this parameter. One of the advantages of our proposed method is that it will eliminate the need for a lateral lumbopelvic X-ray, which has significantly fewer indications to be performed rather than an AP X-ray. Accordingly, it will reduce the patient’s exposure to radiation, and it lowers the costs imposed on the health care system. Consequently, our proposed method can enable us to conclude more objective and anatomical-based information from an AP radiograph image.

Recently a new learning-based method was proposed to regress the pelvic tilt angle from a radiograph image by using convolutional neural networks (CNNs) and conducting simulation experiments using digitally reconstructed radiographs (DRRs) [13]. This research showed the possibility of eliminating CT acquisition for tilt estimation tasks. They reported an estimation accuracy of 3.22 ± 2.18 deg, but when the model trained on DRRs was used for real images, the estimation error increased because the synthetic images were not sufficiently similar to real radiography images. To overcome this problem, Jodeiri et al. [14] proposed a method to train the system with real radiography images rather than DRRs and address the dataset size limitation by employing transfer learning and data augmentation techniques. Their proposed method consisted of two main steps: First, the Mask R-CNN framework [15] was employed to segment the pelvic shape from the background in the radiography images. Then, following the segmentation network, another convolutional network regressed the PT angle. Their cascaded Mask R-CNN and PT estimation networks utilizing multi-task learning, transfer learning, and data augmentation techniques were capable of estimating the PT with 4.04° ± 3.39° error.

In recent years, the field of medical image analysis has witnessed significant advancements, particularly in the domain of segmentation networks with deep encoder architectures [16]. The integration of powerful deep learning models has revolutionized the accuracy and efficiency of image segmentation tasks [17]. Noteworthy examples include VGG-UNET [18], ResNet-UNET [19], VGG-LinkNet [20], and ResNet-LinkNet [21], which employ deep encoders to extract intricate hierarchical features from medical images. These segmentation networks leverage the capabilities of established deep architectures, such as VGG and ResNet, in tandem with specialized decoding components like UNET and LinkNet. The utilization of deep encoders enhances the networks’ ability to capture nuanced patterns and subtle details in medical images, thus facilitating precise segmentation outcomes. In this context, our work builds upon the advancements in segmentation networks with deep encoders, presenting a novel concurrent learning approach for the estimation of pelvic tilt from anterior–posterior radiographs. Through this innovation, we aim to contribute to the evolving landscape of medical image analysis and further enhance the pre-planning factors for procedures like total hip arthroplasty (THA).

**Figure 1:**
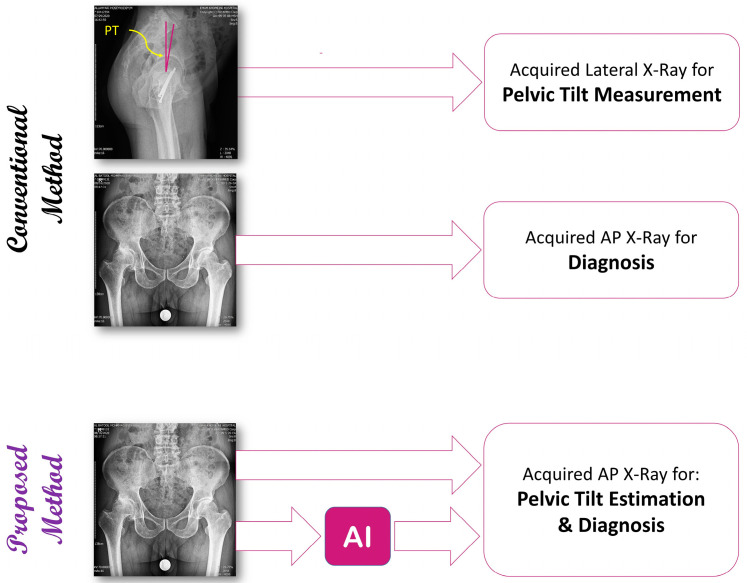
Overview of the registration-based and learning-based methods.

The purpose of this work is to utilize deep learning-based approaches to estimate the pelvic tilt from AP radiographs and hence eliminate the need for further images such as lateral radiographs (Figure 1). This research will open up new possibilities to recognize individual dynamic changes in pelvic rotation accurately with a minimum number of radiograph acquisitions.

The contribution of this paper is threefold: (1) Presenting a novel multi-task encoder–decoder-style network by employing a VGG backbone as a powerful feature extractor for both tasks, including pelvic segmentation and PT estimation. (2) Investigating the advantage of pair-related task learning for more accurate prediction of the main task by employing different architectures for training and testing. (3) Comparison of the diagnostic accuracy of the proposed network and expert surgeons.

In this section, we provide a brief overview of the structure of our manuscript to guide readers through its content. We commence with a background discussion, delving into the significance of accurate pelvic tilt estimation in the context of THA pre-planning. Subsequently, we introduce our proposed methodology in the Methods section, detailing the innovative concurrent learning approach based on VGG-UNET for precise pelvic tilt (PT) estimation from standing anterior–posterior (AP) radiography images. The Results section presents a comprehensive analysis of our approach, comparing it with alternative methods such as VGG and Mask R-CNN. Discussion and Conclusions follow, providing insights into the experimental outcomes and the implications of our findings. We believe that this structured presentation will enhance the accessibility of our work, allowing readers to navigate through the manuscript with clarity and gain a comprehensive understanding of our contributions.

## 2. Materials and Methods

In this section, firstly, the preparation processes of radiography images and corresponding masks and PT labels are explained. Then the main parts of our proposed method, including the U-Net segmentation network, VGG architecture, and constructing a new multi-task encoder–decoder-style network using VGG as its deep feature extractor and light-weight convolutional network as its PT estimator, are described. Finally, we outline how using two different structures in training and testing will enable the system to obtain a more accurate estimate of pelvic tilt.

### 2.1. Dataset Acquisition and Preparation

Our dataset comprises a comprehensive collection of medical records obtained from a total of 180 patients undergoing total hip replacement surgery across three Iranian hospitals: Imam Khomeini Hospital Complex, Sina Hospital, and Shohada Hospital, contributing 126, 47, and 7 cases, respectively. For each patient, the dataset includes essential information such as age, gender, and pelvic tilt (PT). An analysis of the dataset reveals the following statistical characteristics:

- Age: The mean age of the patients is 41.8 years, with a standard deviation of 14.1.
- Gender: The dataset is approximately evenly distributed, with 54.4% male patients.
- Pelvic tilt (PT): The mean pelvic tilt is 3.47, with a standard deviation of 3.10.

**Figure 2:**
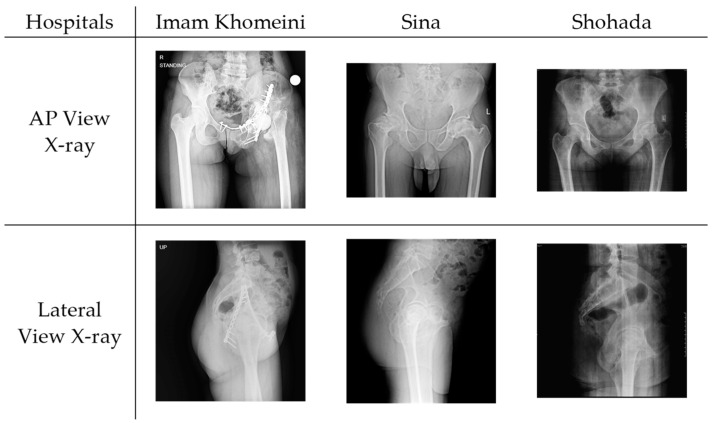
Sample dataset collected from Imam Khomeini Hospital Complex (126 cases), Sina Hospital (47 cases), and Shohada Hospital (7 cases). Images demonstrate the variety in pelvic tilt among patients.

To provide a representative insight into the dataset, Figure 2 below displays a sample of collected data from each hospital. The visual representation includes lateral and anterior–posterior radiography images in the standing position, illustrating the diversity in pelvic tilt across various cases. 

### 2.2. Segmentation Network

U-Net is one of the popular convolutional networks for fast and precise image-to-image translation tasks such as image segmentation and image de-noising [22]. It consists of three general parts, which are encoder, decoder, and skip connections. U-Net passes the feature maps from each level of the encoder over to a similar level in the decoder. In the encoder, local and structural features are extracted. The input size of the image is reduced through the encoder path in order to increase the receptive field, make the model robust to noise and artifacts, and also decrease the computational cost. Increasing the receptive field leads to propagating global information in both time and frequency domains. The skip connections allow the U-Net to consider features at various scales by combining local and global feature maps. In fact, the encoder path captures the context of the input image, and the decoder path extracts the abstract features. Moreover, the precise localization between symmetric feature maps is performed via skip connections.

The capability of the U-Net to learn from a relatively small dataset made it a suitable choice in dealing with medical images, whereas manual preparation of the masks is a very costly procedure. Typically, using a pre-train model to avoid the over-fitting issue in the training of deep convolutional networks seems necessary. In this case, firstly, the network weights are initialized on relatively large datasets with millions of images such as the ImageNet public dataset [23], and are then transferred to the target dataset. Several studies have shown that U-Net can efficiently train from scratch and converge very fast without the need for a pre-train model [24].

### 2.3. VGG Architecture

The main contribution of the VGG [25] was to reveal the impact of the convolutional network depth on the performance of the network in large-scale image localization and classification tasks. The networks before VGG used a large kernel size such as 7 × 7 and 11 × 11 to increase the receptive field, but VGG showed that a sizeable receptive field can be achieved by increasing the depth of the convolutional layers with a 3 × 3 kernel size leads to significantly higher accuracy.

**Figure 3:**
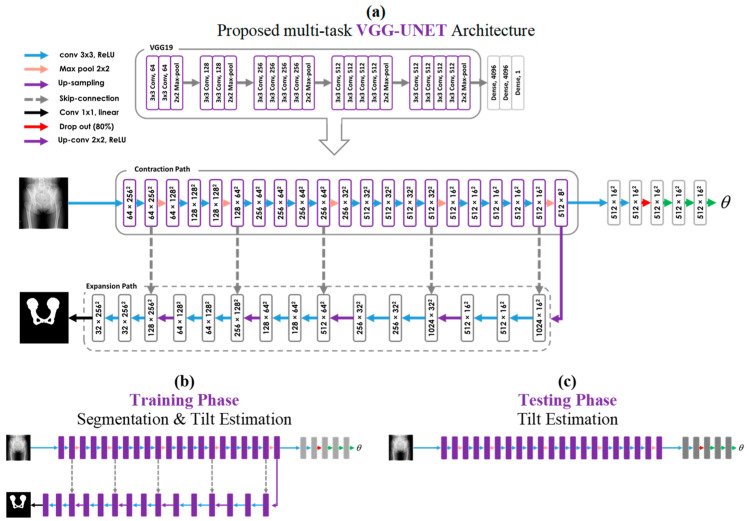
Architecture of the proposed method in the training and testing phases. (**a**) Detailed architecture of the VGG19 and the proposed multi-task system named VGG-UNET. (**b**) Structure of the network in the training phase for learning both tasks, i.e., segmentation and regression. (**c**) Structure of the network in the testing phase for performing the target task, i.e., PT regression.

As shown in Figure 3a, VGG19 consists of 16 3 × 3 CNN layers in five convolutional blocks for feature extraction and three fully connected layers for classification. The first CNN produces 64 channels, and then, at each convolutional block as the network deepens, the number of channels doubles until it reaches 512. All CNN layers are equipped with Rectified Linear Unit (ReLU) non-linearity. Additionally, spatial pooling is carried out by five 2 × 2 max-pooling layers, such that each pooling follows one convolutional block and reduces the size of the input feature map.

### 2.4. Proposed Multi-Task Learning Model

In several studies, it has been demonstrated that the representation depth is beneficial for improving the performance of the system [14]. Motivated by that, we propose a novel encoder–decoder-style network (VGG-UNET) for the pelvic segmentation task. As shown in Figure 3a, to construct the encoder part, we used the five convolutional blocks of the VGG19 as a potent deep feature extractor. The latest extracted features are fed to the decoder part. In the decoder, a 2 × 2 up-sampling and two 3 × 3 CNN layers with ReLU non-linearity are applied, and then this sequence is repeated five times. Contrasting the encoder path, the number of channels in each sequence of the decoder path is halved, eventually reaches 32, and then is converted to binary mask images via one 1 × 1 CNN with a linear activation function. It is notable that, after each up-sample layer, the same-size corresponding tensors in the encoder and decoder parts are concatenated.

The stack of convolutional layers in the backbone of the VGG-UNET is capable of extracting the local features from input radiography images via learnable kernels and non-linear activation functions. In the forward pass, each convolutional layer receives the last feature map and extracts deeper and more semantic features. The richness of the extracted features is maximized in the last convolutional layers of the VGG, i.e., the bottleneck of the U-Net. In the bottleneck, in addition to the decoder path, we used another path utilizing some convolutional and fully connected layers to combine all extracted feature maps from the final convolution layer of VGG and thus regress the PT. As shown in Figure 3a, the network consisted of six layers, including three convolutional layers, a dropout layer, and two fully connected layers. All convolutional layers had 3 × 3 kernel sizes, stride 2, and Rectified Linear Unit (ReLU) activation functions. The numbers of the feature maps for the three convolutional layers were set to 8, 16, and 32, respectively. Following the convolutional layers, 50% dropout regularization and a fully connected layer with 8 Leaky ReLU neurons were used.

#### Multi-Task Training and Single-Task Testing Strategy

Train strategy (Figure 3b) includes concurrent learning of pelvic mask segmentation and PT estimation. For *N* number of samples, Binary Cross-Entropy and Mean Square Error used as a loss function for segmentation and PT estimation, respectively, are defined as follows:

(1) $$Loss_{seg}=-\frac{1}{N}\sum_{i=1}^{N}y_{i}.log(p\left(\hat{y_{i}}\right))+\left(1-y_{i}\right).log(1-p\left(\hat{y_{i}}\right))$$

(2) $$Loss_{reg}=\frac{1}{N}\sum_{i=1}^{N}{\left(y_{i}-\hat{y_{i}}\right)}^{2}$$

where $y\;\;\mathrm{and}\;\hat{y_{i}}$ are the ground-truth and predicted values, respectively. The overall loss function is defined as follows:

(3) $$Loss_{total}=(w_{seg}\times Loss_{seg})+(w_{reg}\times Loss_{reg})$$

where $w_{seg}$ and $w_{reg}$ denote the weights for segmentation and regression losses, respectively. Based on several experiments, we considered both weights to be the same and equal to one.

In the test phase (Figure 3c), we excluded the decoder path and just considered the single target task, i.e., PT estimation. In fact, it did not matter to us if the network could accurately detect the pelvic mask; instead, we wanted to appraise the network about the characteristics of each input image mask to use it in the feature extraction process. So, our proposed network would be able to estimate the PT directly from the radiography image, whereas pelvic shape information was coded in the feature maps during the concurrent learning process. In other words, we informed the network about the importance of pelvic bone segmentation in the tilt estimation task but did not limit it to estimating the PT only from the segmented parts. By learning directly from the raw image, the network would be able to use other parts of the radiography image, such as the shape and angle of the femur, to improve its performance, while their relationship to the PT was unclear for us. Also, the low computational cost and high training and testing speed of the proposed method made it applicable to real-time applications.

The amount of data required for deep learning depends on the complexity of the problem and the dataset variation. When dealing with medical images, a high-complexity problem is accompanied by a small dataset. An effective technique to solve this problem is transfer learning, where the network is initially pre-trained with large non-medical images and then fine-tuned on the target dataset [26]. In this paper, the VGG network, which is used as an encoder and feature extractor, was pre-trained on the ImageNet dataset [23].

## 3. Experiments

### 3.1. Evaluation Metrics

To evaluate the accuracy of PT estimation, the mean absolute error (MAE) was used to measure the difference between true and predicted values. In Equation (2), the terms $y\;\mathrm{and}\;\hat{y_{i}}$ denote the ground-truth and predicted values, respectively, and N is the total number of samples of the test dataset.

(4) $$MAE=\frac{1}{N}\sum_{i=1}^{N}\left(y_{i}-\hat{y_{i}}\right)$$

Based on the MAE values, we categorized PT estimation results into three distinct groups, including perfect, acceptable, and weak prediction, corresponding to $MAE\leq 3^{{}^{\circ}},\;3^{{}^{\circ}}\leq MAR\leq 6^{{}^{\circ}},\;\mathrm{and}\;MAE>6^{{}^{\circ}}$, respectively.

### 3.2. Performance Analysis

We conducted the following two experiments for a detailed analysis of the performance and properties of the proposed multi-task network (VGG-UNET).

1. Single-task network with learning only on the target task (VGG): The proposed network for use in the test phase, which consists of a VGG network and a light-weight convolutional network on top, was used for both training and testing. Regardless of segmentation, a deep network was used for direct learning to see if segmentation as a secondary task could be beneficial.
2. Cascaded networks with independent learning (Mask R-CNN): This experiment is meant to compare the proposed method with the state-of-the-art method [14] for PT estimation from a single radiography image. It has been shown that employing the Mask R-CNN instead of the standard U-Net for pelvic segmentation improves efficiency in terms of the Dice coefficient. So firstly, the Mask R-CNN framework was employed to segment the pelvic shape from the background in the radiography images. Then, following the segmentation network, another convolutional network regressed the PT angle. All networks’ structures and hyper-parameters were set according to the original paper. For convenience, we refer to the cascaded network as its segmentation network name, i.e., Mask R-CNN.

In all of the experiments, the proposed network was optimized by minimizing the Adam algorithm with a 0.0002 learning rate for 600 epochs. At each epoch, a mini-batch size of 16 images was randomly selected from the training images and fed into the network to learn the parameters. We used the Linear and Sigmoid functions for regression and segmentation tasks, respectively. The activation function used for the rest of the network was the Exponential Linear Unit named ELU that tends to converge cost to zero faster and produce more accurate results in comparison to other activation functions. The ELU is defined as follows:

(5) $$ELU\left(x\right)=\left\{\begin{array}{cc} x & x\geq 0\\ \alpha \times (e^{x}-1) & x<0 \end{array}\right\}$$

where *x* denotes the input to the activation layer. The ELU activation layer is equal to the input if *x* is positive and becomes smooth slowly until its output equals -α for negative inputs. The α is set to 1.

In the training process, a random transformation was applied to the images and the corresponding masks to generalize the model’s capability for dealing with new images. For image transformation, random rotation in ±20°, random scaling (0.8:1.2), random horizontal and vertical translation in the scale of ±0.2, and horizontal flipping were applied. It is notable that the same data augmentation was used for all experiments.

To ensure the robustness of our results and establish their independence from the choice of the test dataset, we implemented a five-fold cross-validation strategy with a stratified approach based on pelvic tilt. In this method, the original dataset was randomly partitioned into five equally sized subsamples. During each iteration of the cross-validation process, one subsample was set aside for testing the model, another was designated for validation, and the remaining three subsamples were employed for training both the segmentation and pelvic tilt estimation networks. The use of a stratified approach ensured that each fold maintained a proportional representation of different pelvic tilt angles. This iterative, stratified five-fold cross-validation process was repeated five times, ensuring that each subsample was utilized exactly once as the test data. By incorporating stratification, we aim to address potential biases and enhance the robustness of our model evaluation, contributing to the reliability and generalizability of our findings.

To confirm the fact that accurate estimation of the pelvic tilt from AP radiography is difficult, and in most cases, we might say impossible for the surgeons, we asked 10 experienced surgeons to classify 20 AP radiographs as anterior (A), posterior (P), or unknown (U) tilt. Accordingly, all the images were assigned to the proposed network, and that specific network classified the pelvic tilt for each image as either anterior or posterior tilt. The results of this experiment were compared and analyzed with each other and with their ground-truth values.

The experiments were conducted on a workstation with a Core i7 processor, 3.4 GHz CPU, 8 GB RAM, and Nvidia GeForce GTX 1060 GPU using the TensorFlow library [27].

## 4. Results and Discussion

### 4.1. Comparison with Single-Task Network

**Figure 4:**
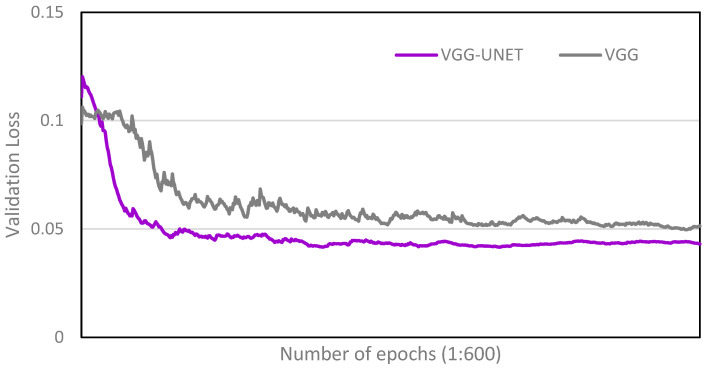
Validation loss for VGG-UNET and VGG.

Figure 4 shows the validation loss function of the VGG-UNET and VGG as the number of epochs increases from 1 to 600 during the training process. This result confirms that the multi-task model learns specific patterns of the pelvic mask from the input images, which are relevant to the pelvic tilt and lead to a significantly lower validation loss.

**Figure 5:**
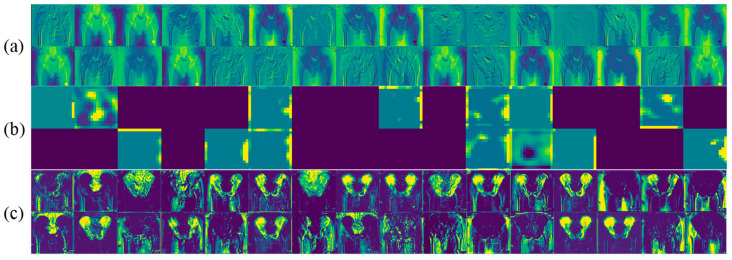
Visualization of the intermediate activations given a specific radiography image: (**a**) sample activations in the encoder path, (**b**) sample activations in the bottleneck, and (**c**) sample activations in the decoder path.

Figure 5 shows the visualization of the intermediate activations given a specific radiography image. It displays the sample feature maps that are produced by the convolution layers in the (a) encoder, (b) bottleneck, and (c) decoder. It seems that the first layers retain all local and structural information by applying various edge detections. There are also some channels of the first layer activation showing the general shape information obtained by applying low-pass filters. As the network becomes more in-depth, the activations become increasingly sparse, abstract, and less visually interpretable. The sparsity of the activations in the bottleneck feature maps indicates that the pattern encoded by the filter is not found in the input image. In other words, the network encodes the high-level concepts related to the label of the image in very abstracted and low-dimensional feature maps. This is where the difference between the two experiments i.e., single-task and multi-task networks, comes from. In fact, the single-task network learns to identify, extract, and encode the features that only correspond to the pelvic tilt, whereas the coded activations in the bottleneck feature maps of the multi-task network contain pelvic bone information too, which is helpful in PT estimation. Figure 5c shows that the network superbly decodes non-interpretable and coded information and extracts features that depict important components in the radiography image, such as the pelvis, femurs, sacrum, obturator foramen, etc., smartly.

### 4.2. Performance Analysis

**Figure 6:**
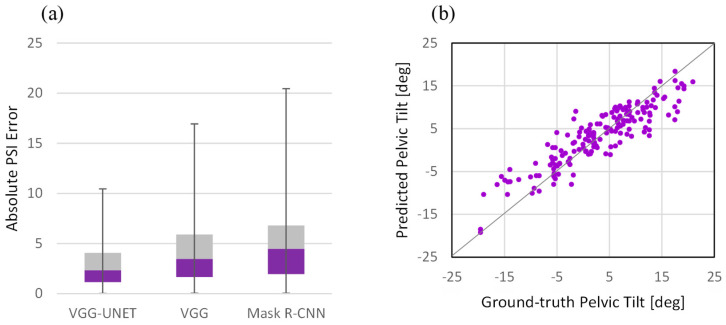
(**a**) Box plot of the absolute PT error for three scenarios, (**b**) scatter plot of the predicted PT as a function of the ground-truth angle for VGG-UNET.

Figure 6 shows a box plot of the absolute PT error for three scenarios and a scatter plot of the predicted PT as a function of the ground-truth angle for VGG-UNET. By comparing the corresponding box of all three experiments, it is intuitively clear that the proposed method outperforms.

In Table 1, we present the performance metrics for various network models, including VGG-UNET, VGG, and Mask R-CNN. The reported values include absolute error, R2 coefficient, and prediction quality. Specifically, the absolute error for VGG-UNET, VGG, and Mask R-CNN is documented as 3.04 ± 2.49, 3.92 ± 2.92, and 4.97 ± 3.87, respectively. Notably, our revised analysis now includes additional networks such as ResNet-UNet, VGG-LinkNet, and ResNet-LinkNet, enriching the comparative insights. Upon careful examination, it is evident that VGG-UNET demonstrates superior predictive accuracy, yielding a lower standard deviation (STD) in comparison to VGG and Mask R-CNN. The R2 coefficients further support the effectiveness of our concurrent learning approach, showcasing the highest values among the considered methods.

Addressing the need for expanded comparison experiments, we have incorporated results for ResNet-UNet, VGG-LinkNet, and ResNet-LinkNet in Table 1. This addition allows for a more comprehensive evaluation, capturing the diverse performance of multiple network architectures. We believe that these enhancements contribute to a more robust assessment of our proposed method for pelvic tilt estimation tasks.

Furthermore, when evaluating prediction quality, VGG-UNET achieves 59.4% accuracy, surpassing VGG and Mask R-CNN by 13.3% and 21.1%, respectively. These findings reinforce the efficacy of our concurrent learning approach, as it consistently outperforms alternative network configurations in terms of both accuracy and precision.

**Figure 7:**
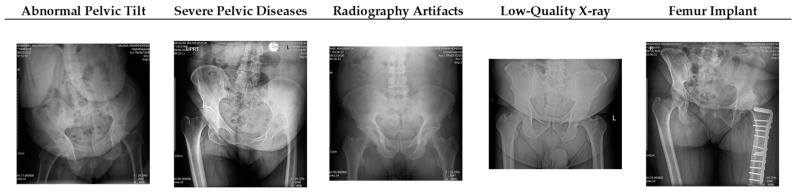
Qualitative examples illustrating challenges encountered by the deep learning model.

To enrich our experimental analysis, we conducted a qualitative investigation focusing on notable examples within our dataset. This qualitative examination aimed to identify patterns of success and challenges faced by our proposed deep learning model. We specifically scrutinized cases involving abnormal pelvic tilt, severe pelvic diseases, radiography imaging artifacts, low-quality X-ray images, and the presence of a femur implant, which were identified as primary contributors to model failures. Figure 7 below presents visual examples of these cases, providing insights into scenarios where the model may encounter difficulties. This qualitative exploration offers a nuanced understanding of the model’s performance across diverse real-world situations, emphasizing its strengths and areas for potential improvement.

### 4.3. Comparison with Surgeon Specialists

For further investigation of the applicability of the AI-based proposed method, we randomly selected 20 patients to evaluate the performance of the proposed network compared to expert surgeons in the diagnosis of standing position pelvic tilt (Table 2).

The results of the comparison of the prediction accuracy of the proposed method with 10 expert surgeons have been reported in Table 2. Overall, eight of the twenty patients had anterior pelvic tilt, and the remaining twelve patients had posterior pelvic tilt. The findings showed that the prediction accuracy of the proposed network for pelvic tilt from AP radiographs was 100%; however, this percentage for surgeons varied from 40% to 60% with an average of 54%, which is significantly less than our proposed network. Approximately, they categorized 30% of the cases as unknown pelvic tilt. In some cases, though, such as numbers 5 and 7, none of the surgeons could diagnose the pelvic tilt correctly. They also had agreement on the prediction of only three cases out of twenty, which indicates that it was a much more challenging issue than they supposed it would be.

As mentioned earlier, the pelvic tilt is representative of pelvic orientation. The pelvis, located at the terminus of the axial skeleton, plays a pivotal role in maintaining sagittal spinal alignment. A pelvic tilt of less than 20 degrees is conducive to promoting favorable spinal sagittal alignment. Additionally, acetabular cup orientation is affected by pelvic orientation; accordingly, pelvic tilt measurement is paramount for patient evaluation for spinal and hip surgery. The present estimation method, lateral spinopelvic X-ray, has disadvantages including extra radiation exposure and its operator-dependent bias.

Finally, it can be concluded that due to the limitation of many devices for standing position imaging and the importance of having accurate, reliable, and sufficient information about the functional pelvis position for successful surgical operation, utilizing artificial intelligence methods can be very helpful.

## 5. Conclusions

This study investigated the applicability of multi-task learning in single-task prediction by using encoder–decoder-style architecture with a deep backbone. Experimental results showed that adding a secondary inter-related task, i.e., pelvic bone segmentation in the pelvic tilt estimation task, improves the performance of the main task significantly.

As mentioned previously, estimation of the pelvic tilt from AP radiographs, even by an experienced surgeon, will lead to about 46% of misdiagnoses. Although T. Schwarz et al. obtained a formula for assessing the pelvic tilt in AP radiographs [28], surgeons’ assessment of pelvic tilt from AP radiographs is not often valid. Nevertheless, the network diagnosed the pelvic tilt with 100% prediction accuracy. Since pelvic tilt influences cup position in total hip arthroplasty, and most patients have some degree of pelvic tilt before their operation, it has to be taken into consideration by surgeons [29]. Moreover, analysis of the pelvic tilt is crucial for the treatment of various orthopedic diseases and hip-preserving surgeries [28,29,30]. All these statements necessitate an accurate measurement of this key parameter with minimum errors. As our proposed AI network has shown no mistake in estimation, it can benefit surgeons in the future in their daily clinical routine.

We believe that the multi-task model, together with the framework’s flexibility and accuracy, will benefit future research on radiography image analysis. In conclusion, this minimally invasive proposed framework has the potential to be routinely used in clinics and hospitals for more precise and accurate estimation performance.

## Figures and Tables

**Table 1:** Performance metrics comparison for various network models including VGG-UNET, VGG, Mask R-CNN, ResNet-UNet, VGG-LinkNet, and ResNet-LinkNet.

	Metrics	Absolute Error	R^2^ Coefficient	Prediction Quality (%)		
Networks				Accurate	Acceptable	Poor
**VGG-UNET**		**3.04 ± 2.49**	**0.80**	**59.4**	**26.7**	**13.9**
ResNet-UNET		3.22 ± 2.82	0.79	57.1	22.9	20.0
VGG-LinkNet		3.53 ± 3.01	0.78	53.6	31.2	15.2
ResNet-LinkNet		3.67 ± 2.52	0.78	49.7	25.6	24.7
VGG		3.92 ± 2.92	0.77	46.1	28.9	25.0
Mask R-CNN		4.97 ± 3.87	0.77	38.3	30.6	31.1

**Table 2:** Comparison of diagnostic accuracy of the proposed network and ten surgeons with the ground-truth values for 20 sample radiography images.

Img#	Ground-Truth		Network Prediction		Surgeons’ Prediction									
					1	2	3	4	5	6	7	8	9	10
1	A	(−6.3)	A	(−3.5)	P	U	U	A	A	U	U	U	A	U
2	A	(−2.8)	A	(−2.8)	A	A	A	A	A	U	U	P	A	A
3	A	(−9.8)	A	(−10.1)	A	A	U	U	A	A	P	U	A	A
4	P	(+4.7)	P	(+5.1)	A	P	U	U	P	U	P	U	U	P
5	A	(−4.2)	A	(−0.2)	P	P	U	P	P	U	P	U	P	P
6	A	(−14.4)	A	(−10.4)	A	A	A	A	A	A	A	A	A	A
7	P	(+4.5)	P	(+4.0)	A	U	A	A	A	A	A	A	A	A
8	P	(+1.2)	P	(+4.9)	A	A	A	A	A	A	A	A	A	A
9	A	(−5.8)	A	(−2.2)	A	A	A	A	U	A	A	A	U	U
10	P	(+6.0)	P	(+9.7)	A	A	A	P	U	U	U	U	P	U
11	A	(−4.9)	A	(−3.7)	A	A	A	A	A	A	A	A	A	A
12	P	(+6.2)	P	(+10.0)	U	U	P	U	P	U	P	U	P	P
13	P	(+5.8)	P	(+7.6)	P	P	P	P	P	P	P	P	P	P
14	P	(+18.0)	P	(+14.5)	P	P	P	P	P	P	P	P	U	U
15	P	(+5.5)	P	(+8.8)	P	P	P	P	U	P	U	P	P	P
16	A	(−5.4)	A	(−6.7)	A	A	U	N	U	A	A	U	A	U
17	P	(+13.6)	P	(+13.5)	P	P	P	U	P	P	P	P	P	P
18	P	(+11.3)	P	(+10.0)	U	U	P	U	U	U	P	U	U	U
19	P	(+4.3)	P	(+8.3)	U	U	P	U	U	P	P	U	P	P
20	P	(+10.3)	P	(+10.7)	P	P	P	P	P	P	U	P	U	U
**Prediction Accuracy (%)**			**100**		**55**	**60**	**60**	**50**	**55**	**55**	**55**	**40**	**60**	**50**

## Data Availability

Dataset available on request from the authors.
